# A systematic review of the blockchain application in healthcare research domain: toward a unified conceptual model

**DOI:** 10.1007/s11517-024-03274-x

**Published:** 2025-01-10

**Authors:** Seyma Cihan, Nebi Yılmaz, Adnan Ozsoy, Oya Deniz Beyan

**Affiliations:** 1 Scientific and Technological Research Council of Turkey (Tubitak), Ankara, Turkey; 2 Hacettepe University Graduate School of Science and Engineering, Ankara, Turkey; 3 Computer Engineering Department, Hacettepe University, Ankara, Turkey; 4 Faculty of Medicine and University Hospital Cologne, Institute for Medical Cologne, Cologne, Germany

**Keywords:** Blockchain technology, Healthcare research, Conceptual model, Clinical trials, Systematic literature review

## Abstract

Recently, research on blockchain applications in the healthcare research domain has attracted increasing attention due to its strong potential. However, the existing literature reveals limited studies on defining use cases of blockchain in clinical research, categorizing and comparing available studies. Therefore, this study aims to explore the significant potential and use cases of blockchain in clinical research through a comprehensive systematic literature review (SLR). To thoroughly investigate all aspects of the subject, we analyzed primary studies based on research questions (RQs) and developed a unified *conceptual model* using *step-based model creation*. Studies from 2015 to 2023 were reviewed, and 34 primary studies were comprehensively analyzed by using the PICO template. In our findings, *privacy* emerged as the most frequently cited requirement in clinical research. The most mentioned use cases for blockchain are *ensuring data immutability* and *security*. A significant issue identified beyond the common blockchain limitations of capacity and scalability is the *lack of standards for compliance with legal frameworks* like GDPR and HIPAA. After all these efforts, we developed a conceptual model, which, to our best knowledge, is the first in the literature to support software developers and clinical researchers in developing and using blockchain-based research platforms efficiently.

**Graphical abstract:**

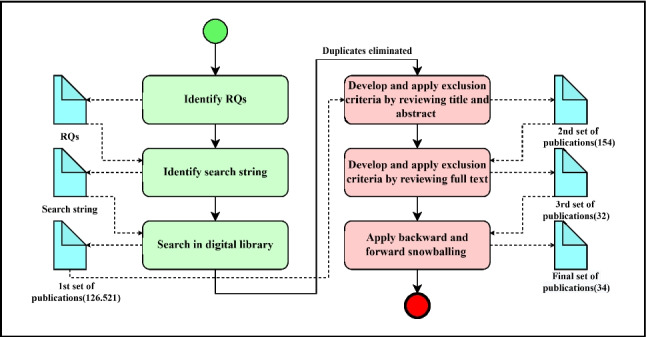

## Introduction

Clinical research plays a crucial role in assessing the effectiveness and safety of new drugs, medical equipment, and healthcare interventions and establishing estimations about the cause and effect of diseases [1]. Although clinical research is vital for advancing medical knowledge and improving patient care, it also presents many challenges. Juneja and Banga [2] outlined these challenges in current clinical research approaches, categorizing them into significant areas such as data management, data sharing, security concerns, and the subject enrollment process. Furthermore, Choudhury et al. [3] highlighted the complexities of multi-center clinical research, emphasizing risks such as centralized data loss and difficulties in regular data updating and integration. Another critical ethical concern in clinical research is obtaining patient consent [4]. Current practices often fail to manage the patient consent process efficiently, such as inadequacies in obtaining permission for added information, failure to provide consent documents to patients, and inaccurate or outdated consent forms [5, 6]. Moreover, clinical data obtained from multiple resources are often unavailable for further use [7] and not interoperable enough [8]. In addition to these challenges, the transparency issues in the publication process of clinical research pose a significant concern regarding the reliability and scientific efficacy of research findings [9].

When the literature was examined, it was noticed that the challenges mentioned above and the approaches and technologies to be used in solving these problems, particularly data sharing in multi-center research, interoperability, and reusability, were not adequately addressed. The insufficient emphasis on these problems in literature causes significant challenges for the clinical research industry. Neglecting to address these problems can impact research efficiency and its outcomes’ reliability. Methodological issues, ranging from scientific misconduct to fraud, leading to reproducibility deficiencies, are serious problems that compromise the outcomes of clinical studies and undermine research quality [10]. Furthermore, it is essential to recognize that failure to tackle such issues can lead to increased costs for the industry and make research processes more complicated [8]. Therefore, it is crucial to fill these notable gaps in clinical research and address them in a broader literature context. Not only does it lead to more robust scientific findings, but it also fosters advancement within the healthcare domain.

Based on all of these, alternative technologies and approaches are required to address complex clinical research requirements and improve clinical research practices [11]. One such technology that has gained attention for its potential in addressing these challenges is blockchain. Blockchain is a distributed, decentralized, and timestamped ledger system that can record continuously growing data and transactions permanently and unchangeably. In addition, blockchain technology can provide logic to the data-exchanging process through smart contracts and next-generation blockchain frameworks [12–14]. Blockchain technology has enormous potential and a global impact on improving the quality and efficiency of clinical research by enabling it to track, share, and manage research data. Above all, it provides the transparency and privacy essential to clinical research and is required by all parties of the clinical research community, such as patients, researchers, and institutions. Technological capabilities such as ensuring data integrity with cryptographic functions, traceability, and chronological recording of data are other essential functions of blockchain technology for clinical research [10]. It is also worth noting that one of the most important factors in blockchain’s prominence is its ability to meet the diverse needs of clinical research single-handedly. Due to the significant potential of blockchain technology to offer substantial contributions in this domain, we aimed to uncover this enormous capacity and explore the use cases in clinical research by examining the details of the studies conducted in this area through a comprehensive SLR. During the SLR process, studies from the most well-known scientific digital libraries (ACM, Google Scholar, IEEE Xplore, Web of Science, PubMed) and from 2015 to 2023 were searched. Since this technology gained popularity in the healthcare sector around 2015, interest has significantly increased by 2018 and beyond. From this point forward, there was a notable surge in scholarly publications and research efforts exploring blockchain’s potential applications in healthcare [15]. After applying inclusion and exclusion criteria to the initially retrieved studies, 34 primary studies were selected [16]. To comprehensively examine all aspects of the subject, the selected primary studies were reviewed and analyzed using the RQs (Research Questions) we formulated.

To better understand the problem areas identified through this SLR study and the solution approaches offered by blockchain technology, there is a clear need for a conceptual model that encompasses the challenges identified by the research questions and solution strategies for them. This conceptual model should synthesize findings from the literature to provide a holistic understanding of the challenges exposed in clinical research. It should also propose innovative solutions and best practices derived from existing research to address these challenges effectively. By integrating information from multiple sources and stakeholders, this conceptual model can serve as a valuable resource to guide future research and practice in clinical research. Therefore, in this study, a conceptual model has been developed based on the output of the SLR. This conceptual model, to the best of our knowledge, is first in literature and includes the fundamental components of the solution, their relationships, and the overall dynamics within the software.

This research has the potential to benefit two distinct groups especially. The results revealed by this SLR study and the developed unified conceptual model can enable both software developers and clinical researchers who are end users. By providing a clear framework, conceptual models facilitate better communication and understanding among clinical research stakeholders, including software developers, leading to more efficient development and implementation of blockchain solutions. This, in return, enhances clinical research systems’ overall quality and reliability, ensuring secure and transparent management of clinical trial data.

More clearly, the main contributions of our study are as follows:

- Identifying the essential characteristics of available literature.
- Outlining the requirements and specifications of clinical research.
- Overviewing the techniques used to validate/verify results in clinical studies.
- Identifying the use cases of blockchain technology in the healthcare research domain.
- Summarizing the requirements specifications for implementing blockchain technology.
- Sketching the services, standards, regulations, and protocols involving blockchain technology.
- Developing a unified conceptual model, to the best of our knowledge, that is first in the literature based on this study’s results.

The rest of this paper follows this structure: Section 2 briefly explains and compares related studies with this SLR. Section 3 presents the research methodology, including details of the SLR process, RQs, the number of primary studies, exclusion/inclusion criteria, and the data extraction process. Section 4 reports the results of the SLR by analyzing data from the reviewed papers. Section 5 summarizes and discusses essential findings. Section 6 presents the conceptual model development process based on this study’s results. Section 7 briefly explains the potential threat to validity. Finally, Section 8 gives conclusions and suggestions for further research.

## Related works

This section explains the former literature review studies investigating blockchain applications in the healthcare research domain (Table 1). To account for the potential omission of relevant secondary studies by our search string outlined in Section 3b, additional searches were conducted using variations such as “SLR,” “systematic literature review,” “review,” “clinic research,” and “blockchain.” As a result of this search, a systematic literature review exists examining the utilization of blockchain technology within the healthcare sector [17–20] the number of studies focusing specifically on its implementation in the clinical research domain remains significantly limited.

**Table 1:** Comparison of the SLR studies

Study	Search string	Number of primary studies	RQs	Followed guidelines	Conceptual model
[21]	-	8	-		-
[22]	-	-	-	-	-
[23]	-	24	-		-
[24]	(“blockchain” OR “distributed ledger technology”) AND (“clinical trials” OR “clinical trial data management” OR “electronic health records” OR “smart contracts”)	16		PRISMA	-

This insufficiency of research highlights a crucial gap in understanding and harnessing the potential of blockchain technology within clinical research. Given the transformative potential of blockchain in enhancing data integrity, security, and transparency, it becomes increasingly evident that dedicated efforts are required to explore its efficacy and feasibility in clinical research. Addressing this gap through targeted studies and initiatives is essential for unlocking the full capabilities of blockchain technology and leveraging its benefits to advance clinical research practices. Of these studies, Omar et al. [21] conducted a literature study on a total of 8 primary studies to highlight the unique features and advantages of blockchain in clinical research. Researchers have categorized existing literature to understand the function of blockchain in clinical research. Consensus protocols used in the study, perspectives on ongoing efforts to implement blockchain solutions in clinical research, and the main obstacles related to the integration of blockchain solutions are addressed.

Katiyar and Singhal [22] executed a systematic literature review study to describe the uses of blockchain technology in clinical trial contexts, delineate the regulatory prerequisites associated with its implementation, and elucidate the obstacles encountered in its adoption. Within the scope of the study, no research questions or specific guidelines were followed to conduct the study. The study provides an overview of how blockchain technology is applied in clinical trials, focusing primarily on the fundamental aspects of both blockchain technology and clinical trials. It covers various phases of clinical trials, explores blockchain integration in clinical studies, discusses regulatory considerations, and examines the challenges associated with these implementations.

Hang et al. [23] carried out a review study that endeavored to uncover the classification of blockchain technology in clinical research. The study examined 24 primary studies. This classification includes decentralized scenarios and applications, blockchain types, implementation methods, and consensus algorithms. Moreover, the study discussed some blockchain projects in clinical research and their micro implementations.

Zhang [24] performed a systematic literature review study using (Preferred Reporting Items for Systematic Reviews and Meta-Analysis) guidelines with the PRISMA intention of highlighting blockchain advantages for clinical trials. The primary studies included in the survey were comparatively examined in terms of their main findings and practical implications. A total of 16 primary studies were reviewed.

Therefore, they do not engage in the synthesis of evidence that formulates RQs and identifies and synthesizes data related to these RQs. However, while not as exhaustive as SLR studies, they might serve the purpose of raising awareness, and this is the principal rationale behind their inclusion and summarization in this section. In addition, although not as comprehensive as SLR studies, they are believed to guide the use of blockchain technology in clinical research.

Additionally, in Table 2, we provide an overview of possible use cases of blockchain technology in addition to basic contributions to the clinical research domain, which are mentioned in the introduction. It is thought that presenting possible use cases of blockchain technology according to the phases of clinical research will provide a better understanding of the key concepts in the study and conceptual model.

**Table 2:** Use cases of blockchain technology in clinical research

Study plan	[10, 25, 26]	Blockchain technology provides a robust solution by permanently recording all aspects and metadata of the research process, including protocols, hypotheses, analysis methods, and subject selection criteria, all with chronological timestamps
Research subject/patient recruitment	[21, 27, 28]	Blockchain has the potential to aggregate all patient-related data, creating a holistic medical history accessible to investigators through a shared framework. It also empowers patients to have more control over their medical data
Patient consent	[21, 23, 25–27]	Blockchain can improve traceability, accountability, and transparency by providing an immutable record of patient consent; this way, it can be easily assessed whether the clinical study complies with the regulations
Data collection	[25, 26, 28–30]	During the data collection phase, blockchain technology offers a time-stamped and signed recording system for the gathered data
Data analyzing	[10, 23, 26]	Data analysts or investigators in clinical research can analyze patient data using these domain-specific smart contracts on a blockchain network. In this phase, statistical analysis should be time-stamped before the data analysis process is completed
Data sharing	[10, 25, 26]	During clinical research, investigators, research subjects, and study funders can efficiently and securely share sensitive personal data using the blockchain structure. Sharing raw clinical data, statistical analysis plans, and datasets significantly contributes to reproducibility and supports clinical meta-analysis studies
Regulatory issues	[23]	Smart contracts embedded in the blockchain can guarantee compliance with clinical trial regulations and rules and protocols set by regulatory bodies
Publication and reporting phase	[25, 26, 28]	Blockchain technology enables detailed evaluations by granting reviewers access to information such as the research protocol, data, and methods. These capabilities significantly enhance the transparency and accountability of the reporting and publishing process

## Research method

**Fig. 1:**
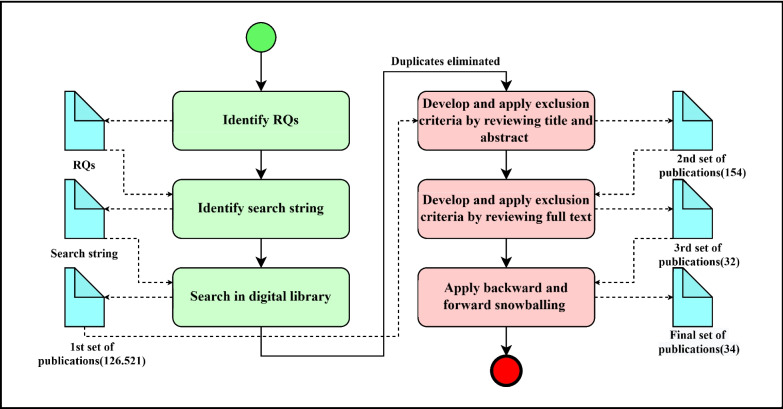
The process of SLR

In this study, the SLR methodology was employed to analyze studies on blockchain applications in the healthcare research domain, adhering to the principles and guidelines outlined by Kitchenham et al. [31]. The steps of this SLR process are shown in Fig. 1. Firstly, we determined the RQs to define the scope of the study. Then, we identified a search string that enabled us to retrieve the most relevant studies from the most known scientific libraries. Then, we ran this search string on digital libraries and obtained the first publication pool. Then, we removed the duplicate studies and applied inclusion/exclusion criteria to the title and abstract of the articles. After this process, we eliminated a set of publications that were not relevant and obtained a second publication pool. Following that, we applied inclusion/exclusion criteria to the full texts of the articles, and after eliminating a set of irrelevant publications, we obtained a third publication pool. Finally, we applied backward and forward snowballing to a third publication pool and obtained two more relevant articles. As a result, we obtained 34 studies to analyze within the scope of our SLR study [16].

- Research question

Formulating suitable RQs is crucial in systematic studies, leading to well-organized and insightful outcomes within a research field. Since this study aims to analyze and classify comprehensively studies on blockchain applications in the healthcare research domain, the RQs are identified in this regard and listed in Table 3. The PICO template suggested by Kitchenham et al. [31] was employed during the identification of the RQs:

- **Population:** Population refers to groups affected by the use of blockchain technology in clinical research. These may include patients, researchers, and healthcare providers.
- **Intervention:** The intervention focuses on implementing blockchain technology in clinical research.
- **Comparison:** The comparison looks at how blockchain performs against traditional methods used in clinical research, such as conventional databases and centralized data management systems.
- **Outcomes:** The outcomes focus on the potential improvements blockchain brings to clinical research.
- **Context:** Academia (scientific literature)

**Table 3:** Research questions

RQ1: What are the essential characteristics of clinical studies in information system literature? RQ1.1 What is the contribution type of the study? RQ1.2 What is the trend in the number of studies over the years? RQ1.3 What are the types of affiliations associated with the authors?
RQ2: What are the requirements and specifications of healthcare/clinical research? RQ2.1 What does clinical research experience the challenges/requirements? RQ2.2 What solutions or use cases does blockchain offer to address these challenges?
RQ3: Have the studies or systems in clinical research been subjected to validation or verification processes? RQ3.1 What methods are employed to validate study data and the system’s performance? RQ3.2 Can the study data and methodologies be reused or repeated? RQ3.3 Have any metrics been determined for the evaluation process?
RQ4: What are the applications of information systems used in clinical studies? RQ4.1 What is the general category of information system/systems used? RQ4.2 What are the requirements or functions associated with using information systems, excluding blockchain?
RQ5 What are the applications of blockchain technology in the healthcare research domain? RQ5.1 Which primary features of blockchain are intended for utilization with health data? RQ5.2 Which blockchain platform was used on clinical research/health data? RQ5.3 Is the blockchain framework permissioned, consortium, or public? RQ5.4 What types of data are stored on the blockchain, and is off-chain or on-chain data storage utilized?
RQ6 What are the requirements for implementing blockchain technology in healthcare research? RQ6.1 What are the strengths and challenges associated with implementing blockchain technology? RQ6.2 What solutions are employed to address these difficulties?
RQ7 What services, standards, regulations, and protocols are used with blockchain technology in the healthcare research domain?

The formulated RQs guided the systematic literature review. RQ1 was determined to be the main profile of the previous studies. This question is important to see what we already have in this domain. RQ2 is one of the questions that most shaped our research. It was created to examine existing clinical research problems and specific blockchain solutions for these problems. RQ3 was constructed to reveal the evaluation process of clinical research. RQ4 was constructed to reveal other technologies used with blockchain technology. RQ5 was formulated to define the application of blockchain technology in clinical research. Along with RQ2, this question (RQ5) is also one of the most important ones shaping this study. RQ6 and RQ7 were designed to investigate the challenges encountered during blockchain application and to explore the services and standards used with it.

b. Search strategy

To retrieve all relevant studies in the literature, searches were conducted in scientific digital libraries utilizing different combinations of search terms, leading to the determination of the following search string:

(“Clinical Research” OR “Clinical Trial” OR “Clinical Studies” OR “Healthcare Research” OR “Healthcare Trial” OR “Healthcare Studies” OR “Medical Research” OR “Medical Trial” OR “Medical Studies”) **AND** (“FDO” OR “Fair Digital Object” OR “FAIR” OR “Findable” OR “Accessible” OR “Interoperable” OR “Reusable”) **AND** (“Blockchain” OR “Block chain” OR “Block-chain”).

Studies within our scope are searched using the provided search string across five academic search engines: Google Scholar, IEEE Xplore, ACM, Web of Science, and PubMed. The number of publications retrieved from the search engines is presented in Table 4. ACM Digital Library yielded the highest number of studies, followed by Google Scholar, among the search engines. Consequently, a total of 126.521 publications were obtained following this search procedure. Medical and general science are covered, including PubMed and other digital resources.

**Table 4:** Search engines

Category	Library	Number of studies
General Science	ACM Digital Library	108.177
General Science	Google Scholar	17.800
Medical Science	PubMed	287
General Science	Web of Science	212
General Science	IEEE Xplore	45

Ultimately, no additional primary studies were acquired beyond our final study pool. Hence, the supplementary search confirmed the adequacy of our search string in identifying primary studies aligned with the objectives of our SLR study.

c. Study selection

Certain inclusion and exclusion criteria were established to ascertain the suitability of the studies retrieved from the digital libraries for our scope. The exclusion criteria (EC) outlined below were identified and utilized to evaluate the first study pool based on their titles and abstracts by following the SLR guideline [31].

EC1: Duplicate studies;
EC2: Studies that are not in English;
EC3: Studies that are not accessible as full text;
EC4: Secondary studies such as systematic literature reviews;
EC5: Not formally reviewed studies such as tutorials, sessions, workshops, keynotes, corrigendum, and panel;
EC6: Books and thesis;
EC7: Studies that focus on blockchain application in healthcare without clinical research.

The following inclusion criteria have been established and considered to ensure that the determined publications align with our scope:

IC1: The study concerns blockchain applications in clinical research.
IC3: The publication year of the study should be between 2015 and 2023.

d. Data extraction

Firstly, basic categories were established to classify publications for RQ, and data extraction was conducted based on these categories. The description of these categories for each RQ will be provided in Section 3. The first and second authors, dividing the tasks between them, thoroughly examined the full texts of the studies to extract data for each RQ. The elicited data were meticulously recorded in a tabular format within a data extraction sheet, utilizing the relevant categories where applicable. When the author identified the emergence of a new data category from a study, it was incorporated into the existing data categories on the sheet following deliberation among the authors. Subsequently, the rest of the authors conducted a peer review of the extracted data by scrutinizing the full texts of the studies.

These authors flagged any conflicts regarding the extracted data on the data extraction sheet. Then, online meetings were conducted with all authors until a consensus was reached regarding these disagreements. Finally, the final version of the data extraction sheet is obtained. This sheet is accessible from the following link [32]. This extraction sheet would be a little inconvenient for aggregating data for readers. Therefore, a document has been created and is available at this reference [33], facilitating readers in identifying the primary study associated with each category related to the RQs.

## Results

This section presents the essential findings and outcomes derived from the literature. By critically examining the literature, the aim is to identify patterns, gaps, and trends, ultimately contributing to a comprehensive understanding of the current state of knowledge on blockchain application in the clinical research domain. The analysis involved a detailed examination of the responses to the RQs. The answers have been categorized and summarized, providing an organized presentation of the findings.

- **RQ1:** What are the essential characteristics of clinical studies in information system literature?
- **RQ1.1** What is the contribution type of the study?

**Fig. 2:**
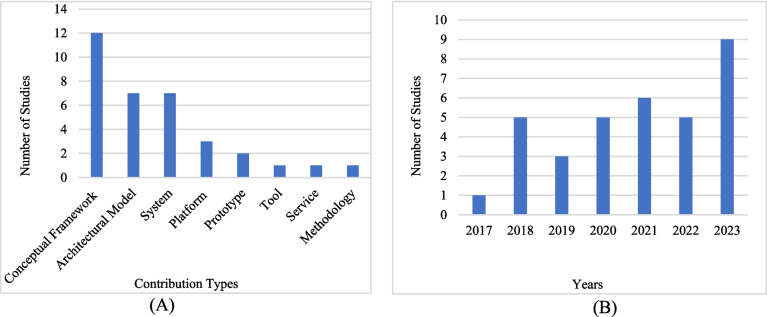
**A** Publication contribution types and **B** publication trends: number of studies concerning years

This RQ examined the study’s contribution type. As shown in Fig. 2A, the majority of the studies have been constructed as a conceptual framework. However, only a small fraction of the studies have been translated into real implementations, and some of them have remained in the prototype stage.

- **RQ1.2** What is the trend in the number of studies over the years?

Figure 2B presents the number of studies by year. Although the research scope spans from 2015 to 2023, academic attention in the field has notably intensified from 2018 onward. In other words, the publication trend has increased in recent years.

- **RQ1.3** What are the types of affiliations associated with the authors?

**Fig. 3:**
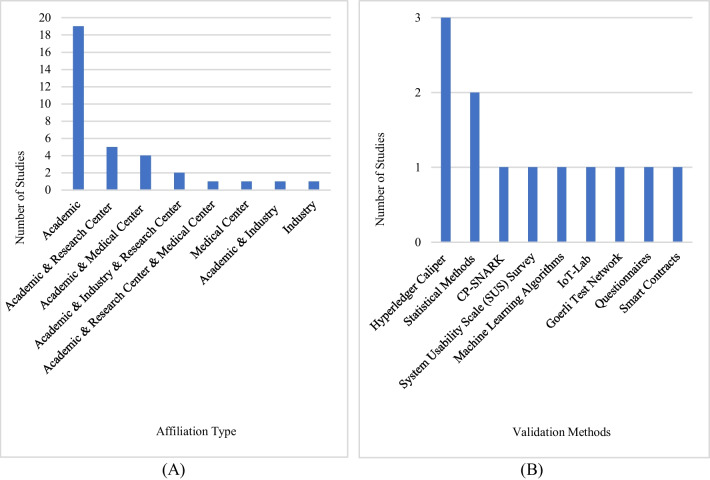
**A** Author affiliation type distribution and **B** distribution of validation methods used in the system

In this RQ, the affiliation types of the authors were examined. According to Fig. 3A, the top three types are 19 studies conducted in academia, 5 with academic and research center collaboration, and 4 with academic and medical center collaboration. In conclusion, studies in this domain have concentrated predominantly on the educational domain.

- **RQ2:** What are the requirements and specifications of healthcare/clinical research?
- **RQ2.1** What does clinical research experience the challenges/requirements?

In the literature, there is a discussion about some challenges and specific requirements of clinical research. These challenges/requirements have been presented in a grouped manner, as shown in Table 5. Accordingly, the most discussed challenge appears to be privacy issues (in 16 studies). Following this, the concerns of security (in 11 studies), data tampering or modification (in 10 studies), medical data sharing among research institutions (in 8 studies), centralization problems (in 8 studies), and data format differences (in 8 studies), are discussed sequentially. Additionally, other findings related to this RQ, such as interoperability, availability, and data quality, which are crucial for establishing a theoretical foundation for the conceptual model, have been provided in detail in Table 5.

**Table 5:** Challenges/requirements of clinical research

Description of challenges/requirements	Frequency
Privacy issues	16
Security concerns	11
Tampering/modification/fabrication/falsification	10
It is challenging to share/exchange medical data among research institutions	8
Centralized database/systems problems	8
Data format/standardization/infrastructure differences	8
Difficulties in recruitment and retention	7
Confidentiality of data	6
Challenging to manage patient consent	6
Regularization barriers	6
Challenges in privacy techniques (differential privacy/anonymization)	4
Transparency issues	4
Reproducibility problems	4
Data quality	4
Reliability of the data/verifiability	4
Traceability concerns/accountability/non-repudiation	3
Reusability of data	3
Selective reporting/publication bias	3
Costly investment and maintenance by participating clinical sites	3
Interoperability	3
Data inconsistency in clinical trials	2
Patient data accessibility	2
Data availability	2
Data integration	2
Digital copyright protection	1
Challenges in data analysis and interpretation	1
Crowdsourcing data collection problems	1
Emerging technologies privacy/security requirements	1
Difficulties related to multi-center study	1
Data fragmentation	1
Data efficiency	1
Data descriptors or metadata requirements	1
Lower level of FAIRness	1

- **RQ2.2** What solutions or use cases does blockchain offer to address these challenges?

The proposed solutions offered by blockchain technology for the challenges mentioned in RQ2.1 have been examined in RQ2.2. Table 6 provides the frequency distributions related to this RQ. The results have been presented by categorizing. Accordingly, the most offered five use cases are ensuring clinical research data immutability (in 21 studies), securing the system (in 13 studies), ensuring the system is auditable and traceable (in 11 studies), allowing patients to control their data (in 9 studies), providing decentralized/distributed architecture (in 9 studies). Table 6 also includes less discussed alternative use case scenarios of blockchain technology in the clinical research domain sequentially.

**Table 6:** Use cases to address challenges and requirements

Use cases to solve challenges	Frequency
Ensure immutable clinical research data/reduce the fraudulent changes/manipulation/modification	21
Make the system secure	13
Ensure the system is auditable /traceable	11
Allow patients to control their data	9
Provide decentralized/distributed architecture	9
Provide privacy/confidentiality of personal data	8
Allows clinical trial data to be transparent	7
Give access control privileges	6
Manage patient consent/re-consent process efficiently	6
Ensure reliable/verifiable data	6
Effective data sharing/exchange networks	6
Address questions of data integrity	5
Managing study protocol/protocol would be time-stamped and transparent	4
Provide interoperability architecture/infrastructure differences can be minimalized	4
Improve the efficiency of study enrollment/recruitment phases	3
Managing the version of the analytical code modifications	3
Provide availability/continuous availability	3
Provide consistency	3
Ensure the completeness of the reporting process	3
Provide accountable data in an open environment	3
Proofs of the authenticity of the clinical trial data	2
Managing study metadata	2
Address questions about data reproducibility	1

- **RQ3:** Have the studies or systems in clinical research been subjected to validation or verification processes?
- **RQ3.1** What methods are employed to validate study data and the system’s performance?

It examines the methods used to validate study data or systems with this RQ. As illustrated in Fig. 3B, three studies validate their proposals by using the Hyperledger Caliper test platform, two by using statistical methods, and the other seven studies by using IoT-Lab, machine learning algorithms, CP-SNARK, the System Usability Scale (SUS) Survey, the Goerli Test Network, Questionnaires, and Smart Contracts to test or validate their study data or system. In the remaining studies, however, no validation method is mentioned.

- **RQ3.2** Can the study data and methodologies be reused or repeated?

This RQ has investigated the reusability and repeatability of the studies. Accordingly, only seven studies have addressed this aspect. One study shared a GitHub repository for reusability. In contrast, others indicated reusability by providing a URL address, requesting information from the author, and referencing the published article as the data source.

- **RQ3.3** Have any metrics been determined for the evaluation process?

**Fig. 4:**
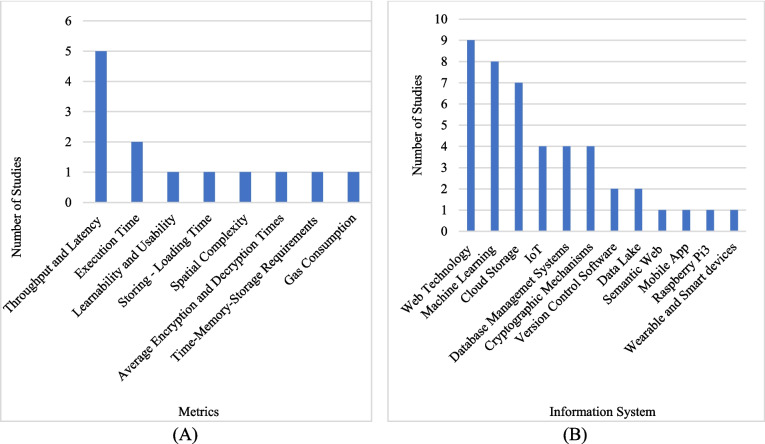
**A** Metrics determined for evaluation process and **B** general category of information system

This RQ examines specific metrics used to evaluate process system performance. Figure 4A illustrates three commonly used metrics: throughput, latency, and execution time. Other less commonly mentioned metrics are spatial complexity, learnability and usability, storage-loading time, average encryption and decryption times, time-memory-storage requirements, and gas consumption.

- **RQ4:** What are the applications of information systems used in clinical studies?
- **RQ4.1** What is the general category of information system/systems used?

It examines which information systems are used to study this RQ. As illustrated in Fig. 4B, when looking at the technologies used with blockchain applications in clinical research studies, Web technology, machine learning, cloud storage, IoT (Internet of Things), DBMS (Database Management System), and cryptographic mechanisms are observed in the forefront.

- **RQ4.2** What are the requirements or functions associated with using information systems, excluding blockchain?

This RQ examines information system usage requirements/functions of studies other than Blockchain. As shown in Table 7, requirements similar to those of blockchain studies are also observed here. When examining the most frequently discussed topics, it is observed that there are Managing clinical study/data, data quality control, data collection, and data access in this context.

**Table 7:** Information system usage requirements/functions in studies other than blockchain

Requirements	Frequency
Managing the clinical study/data	3
Data quality control	2
Data collection	2
Data access	2
Creating case report forms	1
Data security and confidentiality	1
Site selection	1
Patient recruitment	1
Data analysis	1
Accessing up-to-date information	1
Secure design	1
Compliant with regulations	1
Traceable and auditable management process	1
Reduce data errors	1
Data privacy	1
Transparency	1
Collaboration	1

- **RQ5** What are the applications of blockchain technology in the healthcare research domain?
- **RQ5.1** Which primary features of blockchain are intended for utilization with health data?

**Fig. 5:**
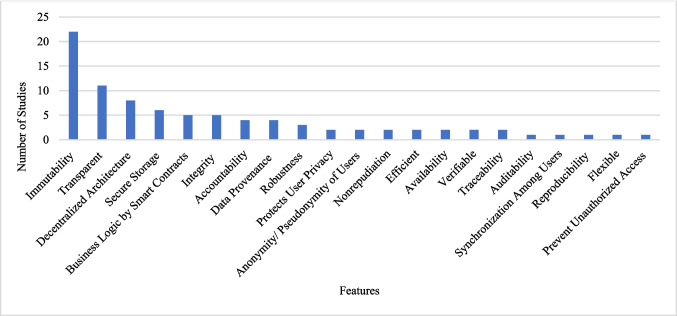
Main features of blockchain

This RQ investigates which main features should be used on health data. Figure 5 depicts the most frequently mentioned features: immutability, transparency, distributed/decentralized architecture, secure storage, integrity, business logic by smart contracts, accountability, and data provenance.

- **RQ5.2** Which blockchain platform was used on clinical research/health data?

**Fig. 6:**
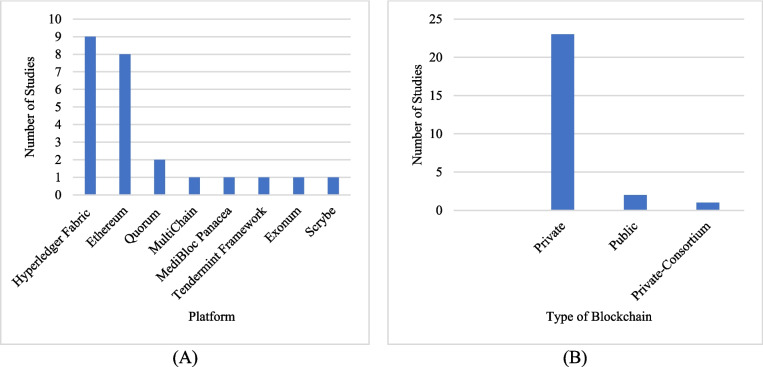
**A** Blockchain platform was used in clinical research and **B** type of blockchain framework

This RQ explores the blockchain platform used in clinical research. As presented in Fig. 6A, the most widely used platforms have been identified as Hyperledger Fabric and Ethereum.

- **RQ5.3** Is the blockchain framework permissioned, consortium, or public?

This RQ analyzes which blockchain framework types should be used for clinical research data. As illustrated in Fig. 6B, the framework identified as the most widely used is private/permissioned blockchain.

- **RQ5.4** What types of data are stored on the blockchain, and is off-chain or on-chain data storage utilized?

**Fig. 7:**
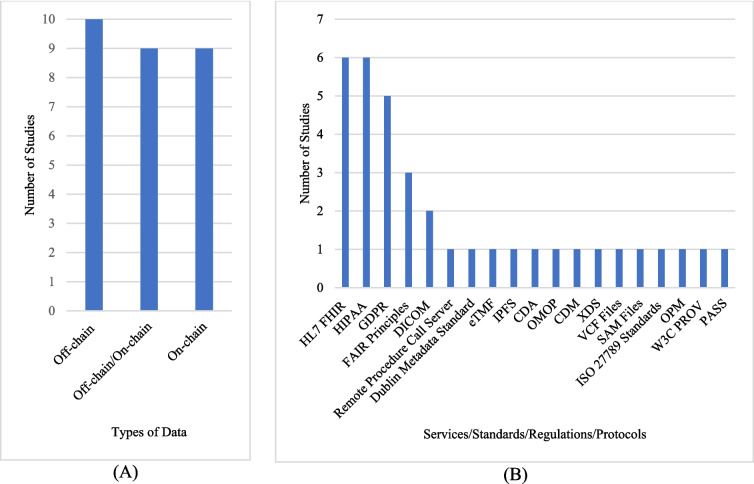
**A** Types of data are kept on the blockchain and **B** services/standards/regulations/protocols used with blockchain

This RQ explores whether off-chain or on-chain data storage is used in clinical research studies. As presented in Fig. 7A, the most frequently used storage type has been explored as off-chain storage. In addition, when examining the type of data stored on the blockchain by studies using on-chain storage, it has been determined that data such as metadata, public key and hash values**,** questionnaire results, quality and authenticity information, and author information were stored. Detailed information regarding this RQ is provided in Table 8.

**Table 8:** Data types stored on-chain

Data types kept on-chain	Frequency
Metadata	2
Public key and hash values	1
Questionnaire results	1
Quality and authenticity information are stored in the form of hashes	1
Author’s information (identity certificate, copyright use, copyright trading)	1
Identifiers and hash codes for data	1
Commitments	1
Annotation	1
Genomic variants	1
Hash values	1
Maternal data	1
Clinical protocol, visit history, subject information	1
Quality control information	1
Patient consents	1
Model contract	
Images	1

- **RQ6** What are the requirements for implementing blockchain technology in healthcare research?
- **RQ6.1** What are the strengths and challenges associated with implementing blockchain technology?

RQ6.1 examines the strengths/challenges of implementing blockchain. Table 9 provides the frequency distributions related to this RQ. A group has presented the results. Consequently, the most debated challenges are the lack of standardized legal frameworks for the utilization of blockchain technology, limitations in scalability, clinical trial management systems demands in terms of throughput and latency, and capacity issues. Table 9 also includes less-considered challenges of blockchain technology in the clinical research domain.

**Table 9:** Challenges of implementing blockchain technology

Challenges of implementing blockchain	Frequency
There is currently a lack of standards compliant with legal frameworks (such as GDPR)	7
Limitations in scalability	6
Clinical trial management systems demand a high transaction throughput and low processing latency	5
Limited capacity of blocks	4
The costly nature of utilizing a private blockchain	2
Remain in the conceptual stage for healthcare application	2
Smart contract usage will require complex interdisciplinary skill sets	2
There is an absence of widely adopted and effectively implemented common data standards	2
Necessitates a certain degree of proficiency in cryptography	1
The Need for Interoperability	1
Healthcare facilities need to cooperate to offer blockchain adapters for system integration	1
The storage space and computational power required by blockchain are greater than those of a centralized database	1
The transaction requires a cryptographic consensus verification	1
Challenges of on-chain data storage	1
Cannot handle the demand for processing large volumes of clinical data in real-time	1
Requires integration with an available clinical database	1

- **RQ6.2** What solutions are employed to address these difficulties?

This RQ examines what solutions are used for the mentioned difficulties in RQ6.1. As depicted in Table 9, the most frequently mentioned solutions have been identified as integrating cloud/decentralized storage for storing extensive medical data, complies with the GDPR, splitting and merging to handle extensive image data to reduce viewing latency, performing parallel processing/sharding to decrease delay, multiple levels of indexing schemes for faster access/querying/analysis data. More results regarding this RQ are provided in Table 10.

- **RQ7** What services, standards, regulations, and protocols are used with blockchain technology in the healthcare research domain?

**Table 10:** Solutions are used for difficulties in the mentioned RQ 6.1

Solutions to addressed the challenges	Frequency
Integrating cloud/decentralized storage to provide off-chain storage for extensive medical data	2
Complies with the GDPR by keeping separate (off-chain data lake) its data from the clinical data (data minimization, right to erasure)	2
Splitting and merging to handle extensive image data to reduce viewing latency	2
Perform parallel processing/ sharding to decrease delay	2
Faster access/querying/analysis with multiple levels of indexing schemes	2
To address the challenges of on-chain data storage by minimizing the data inserted into the chain using reference-based data compression	1
Use an efficient alternative consensus algorithm that is capable of registering provenance for multiple clinical trials	1
Using machine learning technologies to predict the transaction throughput in clinical trials	1
Implementation of the HL7 FHIR API to ensure system interoperability	1
Standards that extend an open protocol that supports efficient standards-based healthcare data exchange	1
Blockchain developers require education on regulatory basics related to healthcare facilities	1
The validation mechanism needed to maintain the scientific integrity of the study	1
The data format must be standardized to promote the interoperability of blockchain solutions for clinical research	1
Smart Contract implementation to improve code consistency across multiple sites	1

In this RQ, an investigation was conducted into the services/standards/regulations/protocols used with blockchain technology in the healthcare research domain. As presented in Fig. 7B, the most frequently used services/standards/regulations/protocols have been determined as HL7 FHIR (Health Level Seven International—Fast Healthcare Interoperability Resources), HIPAA (Health Insurance Portability and Accountability Act) and GDPR (General Data Protection Regulation). Detailed information regarding this RQ is also provided in Fig. 7B.

## Discussion

The primary studies have been thoroughly examined within this study’s scope, considering the RQs, leading to certain conclusions. In this section, the key findings will be discussed and analyzed in the light of secondary studies. Core subjects are grouped in the discussion section to organize and present the analysis readable and cohesive manner.

### Technical implementation

It has been determined that theoretical studies are more dominant than implementation studies (38%), excluding prototyping or experimental simulation testing, in the observed domain (RQ1.1). Blockchain applications are in their early stages, not only in clinical research but also in healthcare in general. Almost half of the studies (42%) show limited implementation maturity levels, such as simulation tests. Most studies are typically in the theoretical or architectural design phase, unable to present real-world implementation or evaluation of their proposed theoretical solutions [17]. As technology evolves and more real-life applications are realized, blockchain will be a strong candidate for becoming the dominant technology in clinical research. However, as of the current state, it is possible to say that the maturity level of this technology has not yet fully reached its potential. Besides, conceptual or architectural design studies have substantial importance in outlining the potential benefits of blockchain solutions for the clinical research domain. However, it is crucial to emphasize that transforming conceptual or architectural designs into detailed conceptual models incorporating specific use cases would be more beneficial for them to guide real-world applications effectively. Moreover, it has also been observed that researchers have employed various definitions in their studies to describe the structures they have developed. Concepts like model, framework, and architecture are used alternatively in primary studies. There is a lack of agreed-upon standards among researchers concerning architectural design. In this regard, adopting a systematic and widely accepted approach is crucial for defining the requirements and the level of detail in outlining the architectural design [34]. To prevent this conceptual ambiguity, it is recommended that guidelines be utilized from professional associations such as IEEE (Institute of Electrical and Electronics Engineers) [35]. In addition, it is considered that the findings of RQ1.3 also support RQ1.1. Many researchers come from the academic field. However, collaborations with research centers, industry, and the medical domain are believed to shift the studies’ focus from the conceptual framework to the implementation axis (RQ1.3).

### Blockchain key use cases

The most mentioned requirement seems to be privacy issues (RQ2.1). This requirement is crucial in the medical domain because protecting sensitive healthcare data is essential [21]. As a concept directly associated with privacy, security is a secondary priority in studies. Serving as a defense mechanism against unauthorized access and usage of personal information, security requirements take precedence in protecting privacy. This highlights the importance of security measures that ensure privacy by protecting critical medical assets. Moreover, providing the ethical conduct of research with human participants requires a strong emphasis on privacy and security. The Declaration of Helsinki mandates researchers engaged in medical research to uphold the confidentiality and security of the personal information of research subjects [36]. Besides, protecting data privacy is a vital requirement for data sharing in clinical trials while making data available for further research [37]. Clinical research often requires data sharing among healthcare organizations. Blockchain technology enhances secure, transparent, and traceable data sharing in clinical research, thereby protecting patient privacy. Moreover, it facilitates secure data transfer between third parties by enabling encrypted data sharing. Its distributed ledger technology allows tracking who shared data, when, and how. This enforces a thrust among clinical research stakeholders while sharing sensitive data. Additionally, sharing raw clinical data and study protocols is crucial in ensuring reproducibility and facilitates clinical meta-analysis studies. When considering other requirements, it was observed that tampering of data, challenges to sharing medical data among research institutions, centralized database problems, data format differences, and difficulties in recruitment and retention phases. It is recognized that the outcomes of RQ2.2 additionally substantiate RQ2.1. The most mentioned use cases for blockchain ensure data immutability and security (RQ2.2). As highlighted by Yaqoob et al. [38], these features demonstrate the key advantages of blockchain technology. The implications of blockchain technology for the field of clinical research are substantial. The crucial ones are that it minimizes the risks of research data breaches and fraud and offers improved security and traceability. Blockchain technology empowers the patient to manage their data. It also supports decentralized data storage to reduce single points of failure. Overall, blockchain can potentially increase the reliability and efficiency of healthcare research results. In this context, the opportunities offered by blockchain closely overlap with the challenges and requirements of clinical research. Blockchain technology can be crucial in addressing clinical research challenges, as mentioned in RQ2.1 [10]. It enhances the reproducibility of clinical research by providing a transparent and immutable record of data transactions. This transparency helps prevent scientific misconduct and fraud, ensuring that research outcomes can be reliably verified and replicated.

### FAIR principles

There was a challenge to a lower level of FAIRness (RQ2.1) (findability, accessibility, interoperability, reusability) [39]. Similar to the examined primary studies, it has been observed that FAIR principles are less common in general healthcare literature. Likewise, there is less awareness and recognition related to FAIRness in clinical research. When data is made FAIR, it addresses many issues related to clinical research challenges revealed in this study, especially in the context of interoperability and reusability. Additionally, to increase the visibility and accessibility of research findings and methodology, although they are missing points in RQ2.1. In addition, FAIR principles ensure data and research output reusability by making them findable, accessible, interoperable, and reusable. They also acknowledge the importance of automated computing in managing data-intensive tasks. Even though mentioned at a lower frequency, data descriptors or metadata requirements have also been stated. The FAIR data principles extend beyond just data to include metadata. In the realm of metadata, attention needs to be given to findability and accessibility prerequisites, whereas achieving interoperability and facilitating reuse demands additional endeavors at the data level [40]. Therefore, it is considered that the implementation of FAIR principles on blockchain technology will significantly strengthen and enhance the solution approaches mentioned in RQ2.2.

### Performance and quality

It became apparent that test platforms like Hyperledger Caliper were used more for measuring the performance of the developed or proposed system, such as transaction speed, latency, and network scalability in the studies (RQ3.1). Therefore, the findings of RQ3.3 also support this conclusion, indicating frequent use of metrics such as throughput, latency, and execution time for the evaluation process. The existing literature, however, offers limited or no recommendations for assessing healthcare applications based on blockchain technology. Assessment processes and metrics should be defined by considering technical and domain perspectives to address this requirement [41] describe healthcare domain-specific assessment metrics such as adhering to HIPAA standards, supporting Turing-complete operations, user identification and authentication capabilities, supporting essential structural interoperability, and supporting patient-centered care. In addition to these findings, it was observed that there is no method specified for the evaluation of validation (RQ3.1), and reusability is expressed at a low rate (RQ3.2); only seven studies have mentioned this aspect; the findings of these two RQs are supportive of each other. It was regarded as provided that a data or system element has been successfully validated; it generally indicates its reliability and compliance with specific standards. This situation can enhance reusability as it increases the likelihood of using this validated data or component in other studies. Additionally, reusable data or components may increase the probability of being verifiable and reliable. In this way, it would also address the reliability and quality requirements (RQ2.1).

### Emerging technologies used with blockchain

It was observed that emerging technologies such as machine learning, cloud storage, and IoT topped the list used with blockchain applications in clinical research (RQ4.1). The advantages of deploying AI-driven blockchain solutions on infrastructure are numerous, fostering flexibility, accessibility, and computational power for onward analytics. The benefits of storing and managing medical data in cloud technologies are illustrated in their function in facilitating medical data exchange. Embracing these technologies initiates a transformation in collaboration, innovation, and enhanced patient outcomes [42–44]. In addition to these aspects, the absence of inherent security measures exposes IoT to privacy and security threats. Blockchain can play a crucial role in addressing essential security requirements in IoT through its security-oriented design. Blockchain’s capabilities can effectively address many structural weaknesses of IoT [45]. Hence, it was opined that it is necessary to establish intermediaries or adapters to govern the utilization of these technologies in a compatible manner that upholds patient data security requirements. Further, the expectations from the information system match the capabilities offered by blockchain (RQ5.1, RQ2.2). Moreover, It was noted that blockchain technology could provide the adequate infrastructure that can independently fulfill numerous functionalities expected from an information system. Additionally, utilizing blockchain technology to build a resilient and trustworthy healthcare information system is a viable alternative to mitigate challenges traditional healthcare information systems encounter, such as data modification, single points of failure, and an absence of data traceability [46].

### Applications of blockchain technology

In a manner resembling [21] and [47], studies of the critical attributes of blockchain technology, including data provenance, transparency, decentralization, and immutability, can assist in addressing rigorous data management challenges in clinical research (RQ5.1). It has been noted that Hyperledger Fabric was widely used. The primary reasons for the widespread preference for Hyperledger Fabric could be considered as factors: efficiency surpasses that of other public blockchains, featuring a modular architecture, a flexible and pluggable endorsement model and consensus algorithms, and a mechanism supporting transaction privacy and integrity through the use of channels (RQ5.2). This allows the creation of channels among distinct member organizations to achieve the concepts of privacy and confidentiality. Additionally, it exhibits lower latency when compared to alternative blockchain platforms [48]. Besides, a multiple-channel approach also can increase transaction throughput by allowing transactions to be performed in parallel. Hyperledger Fabric network stands out not only as an authorized network but also in this aspect [47]. Private/permissioned blockchains are generally used for clinical research (RQ5.3). Unlike the public blockchain, only authorized nodes can join the research network. In this regard, it meets data privacy, the fundamental requirement of clinical research (RQ2.1). The commonly employed storage method has been examined in the context of off-chain storage (RQ5.4). Research has been conducted on alternative storage mechanisms beyond the blockchain system’s on-chain limitations to mitigate computational and resource overheads [47]. One of the challenges posed by using blockchain is the limited capacity of blocks (RQ6.1). Additionally, this parallels with the findings of RQ4.1, where cloud storage technologies are prominently featured among the technologies used with blockchain and among the solution approaches (RQ6.2), integrating cloud/ decentralized storage to provide an off-chain storage solution.

### Compliance with the regulatory framework

It was observed that a notable issue, standing out beyond commonly known limited capacity, scalability, and performance problems of blockchain technology, is the absence of necessary standards for compliance with legal frameworks such as GDPR and HIPAA (RQ6.1). This problem is particularly critical, especially in the context of health research. The inherent decentralization and immutability of blockchain technology, while offering significant potential, present unique challenges when it comes to conforming to strict privacy and security regulations such as the Health Insurance Portability and Accountability Act (HIPAA) and the General Data Protection Regulation (GDPR) [19, 49 have defined a solution approach based on recommendations from studies focusing on GDPR and HIPAA compliance with blockchain. These are keeping sensitive patient data *off-chain* while maintaining only hash values or references on the blockchain, *encrypting patient data* before being stored off-chain, applying smart contracts for efficient *patient consent management*, utilizing off-chain storage and smart contracts to achieve *data minimization*, establishing mechanisms for *data portability* as required by GDPR, *using a private blockchain*, supporting broad spectrum standards and interoperability protocols. The statements indicate that the solutions proposed for GDPR compliance in RQ6.2 are in parallel with these approaches. In addition to compliance with the regulatory framework, these actions enhance privacy in blockchain-based applications, all of which help protect sensitive information from unauthorized access. Furthermore, features such as anonymity, data minimization, and robust user consent mechanisms contribute to maintaining user privacy and fostering trust in these technologies. Moreover, applying FAIR principles specifically endorses the last suggestion of [49]’s study. The RQ7, which focuses on commonly used services and standards, is highly connected to this topic. Generally, HL7 FHIR, HIPAA, and GDPR are mentioned in primary studies. At this point, it can be stated that a less-discussed topic is metadata standards. Specialized metadata standards defined for clinical research can be described and utilized [50] proposed a basic, cohesive metadata framework specific to clinical research based on the DataCite standard. DataCite is a general-purpose metadata standard that identifies and makes research data discoverable online. This standard typically includes unique identifying information for scientific research, datasets, papers, and other research outputs [51]. Working with a metadata standard specific to clinical research is also necessary for implementing FAIR principles, one of the topics identified in RQ7.

### Highlights of our study

Our study stands out for its meticulous examination of the subject matter, guided by *comprehensive research questions* and conducted with a *systematic protocol* unlike previous studies (as seen in Table 1). Additionally, this research provides a much more detailed identification of clinical research requirements and defines blockchain use cases tailored to meet these needs. As highlighted in RQ2.1 and RQ2.2, our classification is grounded in specific use cases rather than general categorizations typically found in previous review studies (as seen in Table 2). This more detailed and *use-case-driven categorization* sets our study apart by offering a deeper, more tailored understanding of blockchain applications in healthcare research. We can provide a refined analysis highlighting blockchain technology’s unique aspects by adopting a granular approach. This enables researchers and practitioners to comprehend the various roles blockchain better can play in addressing clinical research-specific needs. Furthermore, a key distinction of our review compared to existing literature is our focus on identifying and addressing the challenges encountered while implementing blockchain technology. We also offer a comprehensive overview of commonly employed solutions to these challenges. This adds practical value to those looking to adopt blockchain in real-world healthcare settings, particularly clinical trials. This makes the study theoretical and a practical resource that informs how blockchain solutions can be effectively deployed. Additionally, our review uniquely covers the evaluation of validation and verification processes and the specific metrics used during these implementations. The most significant difference of our study is that we designed a *conceptual model* based on the outcomes of this literature review, which, to the best of our knowledge, is the first in the literature to support both blockchain developers and healthcare researchers.

## A conceptual model

In the clinical research domain, there is an evident need for a comprehensive conceptual model that encompasses the challenges revealed by the RQs of this study and provides solution approaches for them based on the related literature. However, this conceptual model should be holistic, guiding at the level of implementation details and encompassing all dimensions addressed by the RQs. In the software aspect, a unified conceptual model typically refers to an abstract representation of software or systems’ fundamental structure and functionality during the design phase. This model encompasses the core components, their relationships, and the overall interaction within the software. Moreover, a unified conceptual model that explicitly outlines and establishes connections among these vital elements to clarify the fundamental concepts of system, model, metamodel, modelling language, transformations, software platform, and software product [52]. A precise necessity for a unified conceptual model arises to facilitate researchers in the healthcare domain with a deeper understanding of the problem areas highlighted by this SLR study and the potential solutions offered by blockchain technology. This model should address the challenges identified by the RQs and provide viable solution strategies grounded in relevant literature. Thus, the present study attempts to introduce such a conceptual model, drawing upon the SLR findings.

### Development process of conceptual model

**Fig. 8:**
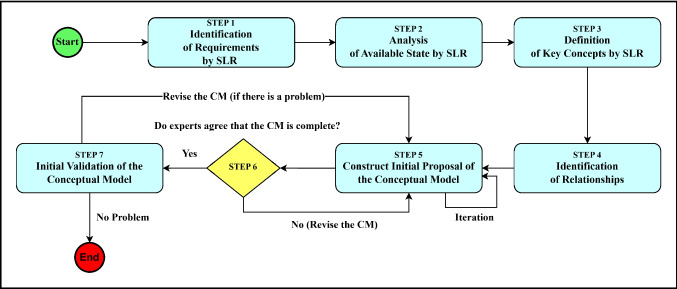
Development steps of conceptual model

This section describes the approach used in developing the conceptual model. The methodology for developing the conceptual model follows an iterative process, as shown in Fig. 8. The step-based process for conceptual model creation, as described in Fig. 8, has been used, which is frequently employed in the literature for developing conceptual models [53, 54]. As can be seen from the figure, the progression of the conceptual model development involves iterative cycles, wherein meta-model concepts undergo continual refinement. This step-based process ensures the systematic and detailed development of meta-models, enhancing their accuracy, usability, and applicability across different domains.

In Step 1, the requirements of the clinical research process were determined for constructing the basic functionalities of the conceptual model. The SLR study guided us to identify these requirements. In Step 2, existing systems were analyzed in detail. This step provides an understanding of the current situation and forms the basis of the modelling process. In this step, the output of this SLR is also utilized. In Step 3, key concepts of the modelling process are identified by thoroughly analyzing existing studies. These concepts often represent important components and modules related to the system. Step 4 determines the relationships and interactions between the identified vital concepts, defining the structure and connections of the conceptual model.

In Step 5, an initial proposal for the conceptual model was formulated, considering the results of Steps 1, 2, 3, and 4. It is important to emphasize that a review-and-revise [55, 56] approach is adopted at this stage. This involved a series of discussions among the authors of this article to evaluate the conceptual model and the development process, followed by necessary revisions. Improvements were also implemented in these sessions. As a result, final decisions were reached through a series of iterations. The output of this step illustrates the fundamental structures and relationships among them. During Step 6, software development experts reviewed the conceptual model, aiming for an external assessment other than the authors. For this purpose, five experts were selected, two from industry and three from academia, each with over 10 years of experience in software architectural modelling and development [57]. Feedback was gathered through semi-structured meetings using predefined questions [58]. A series of online and face-to-face meetings were held in which each expert was interviewed individually, and detailed responses to each question were gathered. On average, each session lasted for 1 h. During these sessions, the experts provided valuable feedback on various aspects of the conceptual model, such as its structure, applicability, and overall coherence. Based on the suggestions of experts, a review-and-revise was applied in several iterations, gradually refining its concepts and relationships. This iterative process continued until a consensus was reached that the conceptual model was comprehensive and applicable. As a result, the conceptual model matured and reached the most optimal version, incorporating all feedback. Finally, in Step 7, the initial version of the conceptual model should be tested in real-life environments. If necessary, revision should also be conducted during this phase. However, this could not be done within the scope of this study. This section is planned to be covered in the future studies.

### The conceptual model

**Fig. 9:**
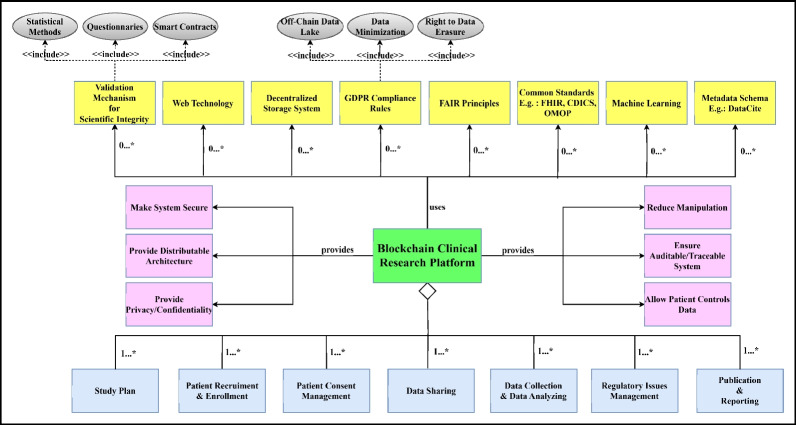
Conceptual model

In a conceptual model, the affordances provided by the system are often specified to describe the system’s functionalities, capabilities, and benefits offered to the target user. This provides a general understanding of the developed system’s design, features, and operation. Table 11 outlines the relationship between RQs and the corresponding segments of the conceptual model. This highlights the interplay between RQs and the structural composition of the conceptual model. Figure 9 shows the conceptual model revealed by the literature review and comprehensive analysis of this study. The modules in blue, located at the conceptual model’s lower edge, express the primary use cases of blockchain currently mentioned in the literature. Blockchain Clinical Research Platform *consists of a study plan, patient recruitment & enrollment, informed consent management, data sharing, data collection & data analyzing, regulatory issues management, and publication & reporting* modules. The pink ones show the modules containing the user benefits we discovered due to our RQs and detailed analysis. Therefore, the blockchain clinical research platform consists of modules such as *make the system secure, provide distributable architecture, provide privacy/confidentiality, reduce manipulation, ensure auditable/traceable system, and allow patients to control data*. Yellow ones indicate the modules or components used to support the features provided by the developed system. This may include elements such as the user interface, integration capabilities with other systems, or capabilities to comply with specific standards and data analysis. Thus, the blockchain clinical research platform *uses validation mechanism for scientific integrity, web technology, decentralized storage system, GDPR compliance rules, FAIR principles, common standards, machine learning, and metadata schema*. Also, *GDPR compliance rules include off-chain data lake, data minimization, and right to data erasure. The validation mechanism for scientific integrity includes statistical methods, questionnaires, and smart contracts.*

**Table 11:** RQs and conceptual model links

RQs	Conceptual model components
**RQ2**	Study Plan, patient recruitment & enrollment, informed consent management, data sharing, data collection & data analyzing, regulatory issues management, publication & reporting
**RQ3**	Validation mechanism for scientific integrity
**RQ4**	Web technology, decentralized storage system, machine learning
**RQ5**	Make the system secure, provide distributable architecture, provide privacy/confidentiality, reduce manipulation, ensure auditable/traceable system, allow patients to control data
**RQ6**	Decentralized storage system, machine learning, FAIR principles, common standards, validation mechanism for scientific integrity, GDPR compliance rules, metadata schema
**RQ7**	FAIR principles, common standards (FHIR, CDICS, OMOP)

The developed conceptual model is thought to provide significant benefits to both developers in the software development process and target users. Our Conceptual Model:

- Can help developers and end users better understand requirements, functions, and relationships. It can also facilitate communication between different stakeholders.
- Can be used to define software requirements. This can help developers and end users define and validate their needs more clearly.
- Can be used to plan and analyze the design of software. This can help developers and designers visualize and analyze system requirements, components, and their relationships.
- Can help developers manage a system from start to finish more comprehensively and systematically and build their applications more quickly.
- Can reduce costs and time in the system development process.
- Can contribute to making overall health systems more effective and reliable by increasing patient safety and research processes’ efficiency in healthcare implementation.
- Can enable more rapid development of appropriate treatment methods and approaches.
- Can lead to a higher quality of healthcare services.

This study reveals the need for a unified conceptual model through RQs and detailed analysis. This conceptual model emphasizes the essential functions and capabilities in the literature, the components that support these functions, and the benefits the system will provide users. This research presents a unified and well-constructed conceptual model for software developers and researchers within the clinical research domain. A robust conceptual model offers a clear and structured guideline that helps bridge the gap between these two groups, facilitating more effective collaboration throughout the software development process. For software developers, the model offers a comprehensive understanding of the specific requirements and challenges in clinical research, enabling them to design and implement more targeted and effective blockchain solutions. The model elucidates blockchain technology’s technical aspects and capabilities for clinical researchers, allowing them to better articulate their needs and expectations. By enhancing communication and mutual understanding among all stakeholders, the conceptual model leads to more efficient development cycles and higher-quality outcomes. Ultimately, this results in clinical research systems that are more reliable, secure, and transparent, ensuring the integrity and confidentiality of clinical research data.

## Potential threat to validity

A systematic approach recommended by Kitchenham et al. [31] is followed during the SLR process, and the step-based process by Beydoun et al. [53] and Othman et al. [54], one of the most widely used in the literature, is employed during the development of the conceptual model. Despite all this effort, there were a number of threats to its validity. Therefore, in this section, these threats are discussed.

Several combinations of search strings are used to perform the searches, and the results are observed in several publication databases. Despite the search string being reviewed and finalized during this process, some studies may still be missed. To mitigate this threat, some complementary search is performed on databases. In this context, snowballing (backward and forward) is employed, the related work section of the primary studies in our study pool is analyzed, and the related work section and primary study pool of the secondary studies discussed in Section 2 are examined.

There is a possibility that the researcher applied the inclusion/exclusion criteria biasedly to eliminate the irrelevant articles. The researchers independently carried out a systematic voting procedure to eliminate this threat using inclusion/exclusion criteria to determine the final publication pool. The publications agreed upon by a majority of authors are included; otherwise, they are excluded. If there is conflict among the authors, it is resolved after a series of discussions. Also, the data extraction process is likely to be affected by researchers’ bias. In order to eliminate this threat, data was extracted by the first two authors. Then, other authors conducted a random peer review to ensure the data was correctly extracted. Furthermore, a non-author independently selected and randomly reviewed publications from the final pool to further validate data extraction. Additionally, the second non-author researcher reviewed publications not selected by the first non-author researcher, thus attempting to increase the accuracy of the data extraction process conducted by the first two authors. This peer review process aims to objectively assess the extracted data and ensure that the findings are reliable and robust. The involvement of multiple independent researchers is thought to increase the research’s reliability and strengthen the integrity of the overall study by reducing potential bias.

Researchers are essential in determining concepts and relationships between these concepts in conceptual models. This may result in overlooked or unused concepts due to the researchers’ bias. To eliminate this threat, a semi-structured interview was conducted with subject matter experts to review the conceptual model. In this context, they reviewed the model and revised it based on their suggestions. Also, the low understandability of RQs (Table 3) may potentially threaten validating the conceptual model. To mitigate this threat, semi-structured interviews are conducted, and the experts are provided with the information they need about the unclear points on RQs or conceptual models.

## Conclusion and future works

Clinical research is indispensable for the development of effective healthcare practices. Therefore, it is critical for developing medical knowledge and improving the quality of patient care. However, it also brings various problems and difficulties, such as data management, data sharing, and security issues. Given these issues, new technologies and methods are needed to meet the complex requirements of clinical research and improve current practices. Blockchain is one of the technologies that has attracted attention due to its potential to overcome these challenges. This paper conducts a systematic literature review that helps achieve its primary objectives. It identifies gaps in the existing literature and discusses areas where evidence is insufficient, conflicting, or under-researched. By addressing these gaps, the study provides valuable guidance for future research.

Extensive literature analysis has shown that blockchain technology can significantly enhance data security and privacy in the healthcare sector, protecting sensitive patient information from breaches. Additionally, it improves data interoperability across healthcare systems, facilitating communication and collaboration among providers, which can lead to better patient outcomes. Moreover, blockchain streamlines clinical trials by ensuring data integrity and participant consent tracking, ultimately accelerating the research process. By giving patients greater control over their health data, blockchain emerges as a powerful technology that fosters interaction and trust among stakeholders, opening the way for a more efficient and collaborative healthcare environment.

The study determined that theoretical blockchain studies in the healthcare domain were more dominant than implementation studies (38%). This shows that the studies are at limited application maturity levels and typically in the design phase. As real-life applications of blockchain are implemented, blockchain will become a stronger candidate in clinical research. Besides, it is thought that there is a lack of agreed-upon standards in terms of defining the structures developed by researchers in their studies. In this context, adopting a widely accepted approach is considered important in defining the requirements and level of detail of the architectural design. Additionally, the most frequently mentioned requirements of clinical research appear to be privacy and security. Providing immutable clinical research data, making the system secure and auditable, allowing patients to control their data, providing decentralized architecture and ensuring the privacy of personal data are the most frequently mentioned use cases. Therefore, the opportunities offered by blockchain were determined to be closely aligned with the requirements or challenges of clinical research. Moreover, in the clinical research literature, awareness of the FAIR principles has been found to be quite low. When data is made FAIR, it will provide solutions to clinical research challenges, especially in terms of interoperability and reusability. Therefore, the application of FAIR principles on blockchain technology will significantly support solution approaches and increase their effectiveness. In addition, it is seen that metrics such as *throughput*, *latency,* and *execution time* are frequently used in the system evaluation process. However, the existing literature is quite limited in terms of evaluating healthcare applications based on blockchain technology. The need to define evaluation metrics specific to the healthcare domain is clearly seen. Moreover, beyond the widely known limited capacity, scalability, and performance issues of blockchain technology, the lack of standards required for compliance with legal frameworks such as GDPR and HIPAA, which are critical, especially in the context of health research, has been identified as a significant challenge in implementation.

Considering all these nested and complex requirements, there was a need for a conceptual model that would make it easier to understand better the problem areas identified by this SLR study and the solution approaches offered by blockchain technology. To address this need, a conceptual model, to our best knowledge, is the first in the literature that was developed using step-based modelling to synthesize findings from the literature and propose innovative solutions and best practices to provide a holistic understanding of emerging challenges in clinical research. Besides, theoretical studies on blockchain technology have significantly dominated the existing literature, as mentioned before. This research aims to bridge the gap between theory and practice by providing a robust conceptual model and clinical research-specific results. It is expected that these findings will not only enrich the current understanding of blockchain applications in the healthcare research domain but also guide further empirical studies. This research is believed to encourage researchers to develop more effective and reliable blockchain applications. The conceptual model also enhances software development by clarifying requirements and functionalities and improving stakeholder communication. It aids in defining and validating software needs, planning system design, and visualizing components, ultimately allowing for a more organized approach that reduces development time and costs.

The developed Conceptual Model needs to be validated in real-world cases. During this validation phase, revisions should be made to the model when deemed necessary. However, such a validation and revision process could not be carried out within the scope of this study. In future research, it is planned to address this important step and evaluate the model’s applicability in the healthcare research field. These evaluations are critical to demonstrating the model’s effectiveness and validity in practice. Therefore, a real-life application will be carried out in future studies by choosing one of the use cases specified in the conceptual model. The conceptual model will be validated with real-world data from clinical research institutes. The expert opinions will be used to validate the experimental results obtained. Since there is no previously developed conceptual model in this field in the literature, expert opinion and case studies will be used for validation purposes. These methods are important in the validation phase of the literature. In addition, using the multiple-embedded case study design [59], our conceptual model will be applied to defense data, one of the domains where data privacy and security are important, and the results will be evaluated by experts. This approach broadens the research findings’ scope, helping achieve more meaningful results.

Moreover, as mentioned before, a key issue recognized beyond the typical blockchain constraints of capacity and scalability is the lack of established standards for compliance with legal regulations like GDPR and HIPAA. Future studies can focus on developing comprehensive standards and best practices that ensure blockchain applications in healthcare and clinical research comply with these regulations. This could involve creating frameworks for data anonymization, consent management, and tracking records, facilitating secure data sharing while maintaining compliance. Ensuring the security of sensitive data while preserving privacy can be a complex administrative task, particularly in clinical settings where personal health information must be protected under strict regulations. Therefore, collaborating with legal experts and regulatory bodies will be essential to align blockchain solutions with existing laws, ultimately fostering greater trust and adoption in the healthcare industry.

## Data Availability

The data that support the findings of this study are openly available on Google Drive and Zenodo at the following URL: 2. Data extraction sheet, Google Drive, URL: https://tinyurl.com/2d4nltak 3. List of primary studies of the SLR, Zenodo, URL: https://zenodo.org/records/12624794 4. Matching articles with categories, Zenodo, URL: https://zenodo.org/records/12624899 5. A List Of Questions For Obtaining Feedback From Experts, Zenodo, URL: https://zenodo.org/records/12624582 6. Expert features consulted on the conceptual model, Zenodo, URL: https://zenodo.org/records/13932035

## References

[1] Inan OT, Tenaerts P, Prindiville SA, Reynolds HR, Dizon DS, Cooper-Arnold K, Califf RM (2020) Digit Clin Trials NPJ Digit Med 3(1):101. 10.1038/s41746-020-0302-y 10.1038/s41746-020-0302-y PMC7395804 32821856

[2] Juneja M, Banga R (2018) Clinical trials on blockchain. PhUSE EU Connect 2018, Toronto, Canada.

[3] Choudhury O, Fairoza N, Sylla I, Das A (2019) A blockchain framework for managing and monitoring data in multi-site clinical trials. arXiv preprint arXiv:1902.03975. 10.48550/arXiv.1902.03975

[4] Nijhawan LP, Manthan D, Janodia BS, Muddukrishna KM, Bhat KL, Bairy N, Udupa N, Prashant B, Musnade (2013) Informed consent: issues and challenges. J Adv Pharma Technol Res 4(3):134–140. 10.4103/2231-4040.116779 10.4103/2231-4040.116779 PMC3777303 24083200

[5] Barney JR, Antisdel M (2013) Common problems in informed consent. Human research protection program (hrpp).

[6] Choudhury O, Sarker H, Rudolph N, Foreman M, Fay N, Dhuliawala M, Sylla I, Fairoza N, Das AK (2018) Enforcing human subject regulations using blockchain and smart contracts. Blockchain in Healthcare Today. 10.30953/bhty.v1.10

[7] Queralt-Rosinach N, Kaliyaperumal R, Bernabé CH, Long Q, Joosten SA, van der Wijk HJ, Roos M (2021) Applying the FAIR principles to data in a hospital: challenges and opportunities in a pandemic. MedRxiv. 10.1186/s13326-022-00263-7 10.1186/s13326-022-00263-7 PMC9036506 35468846

[8] Abdelouahid AR, Debauche O, Mahmoudi S, Marzak A (2023) Literature review: clinical data interoperability models. Information 14(7):364. 10.3390/info14070364

[9] Baskerville R, Vom Brocke J, Mathiassen L, Scheepers H (2023) Clinical research from information systems practice. Eur J Inf Syst 32(1):1–9. 10.1080/0960085X.2022.2126030

[10] Benchoufi M, Ravaud P (2017) Blockchain technology for improving clinical research quality. Trials 18(1):1–5. 10.1186/s13063-017-2035-z 28724395 10.1186/s13063-017-2035-z PMC5517794

[11] Embi PJ, Payne PR (2009) Clinical research informatics: challenges, opportunities and definition for an emerging domain. J Am Med Inform Assoc 16(3):316–327. 10.1197/jamia.M3005 19261934 10.1197/jamia.M3005 PMC2732242

[12] Sotos J, Houlding D (2017) Blockchains for data sharing in clinical research: trust in a trustless world. Intel, Santa Clara, CA, USA, Blockchain Appl. Note, 1

[13] Maslove DM, Klein J, Brohman K, Martin P (2018) Using blockchain technology to manage clinical trials data: a proof-of-concept study. JMIR Med Inform 6(4):e11949. 10.2196/11949 30578196 10.2196/11949 PMC6320404

[14] Zhang P, White J, Schmidt DC, Lenz G, Rosenbloom ST (2018) FHIRChain: applying blockchain to securely and scalably share clinical data. Comput Struct Biotechnol J 16:267–278. 10.1016/j.csbj.2018.07.004 30108685 10.1016/j.csbj.2018.07.004 PMC6082774

[15] Taherdoost H (2023) Blockchain and healthcare: a critical analysis of progress and challenges in the last five years. Blockchains 1(2):73–89. 10.3390/blockchains1020006

[16] Cihan Ş, Yılmaz N. List of primary studies of the SLR. Zenodo. https://zenodo.org/records/12624794

[17] Drosatos G, Kaldoudi E (2019) Blockchain applications in the biomedical domain: a scoping review. Comput Struct Biotechnol J 17:229–240. 10.1016/j.csbj.2019.01.010 30847041 10.1016/j.csbj.2019.01.010 PMC6389656

[18] Soltanisehat L, Alizadeh R, Hao H, Choo KKR (2020) Technical, temporal, and spatial research challenges and opportunities in blockchain-based healthcare: a systematic literature review. IEEE Trans Eng Manage 70(1):353–368. 10.1109/TEM.2020.3013507

[19] Hasselgren A, Wan PK, Horn M, Kralevska K, Gligoroski D, Faxvaag A (2020) GDPR compliance for blockchain applications in healthcare. arXiv preprint arXiv:2009.12913. 10.48550/arXiv.2009.12913

[20] Fatoum H, Hanna S, Halamka JD, Sicker DC, Spangenberg P, Hashmi SK (2021) Blockchain integration with digital technology and the future of healthcare ecosystems: systematic review. J Med Internet Res 23(11):e19846. 10.2196/19846 34726603 10.2196/19846 PMC8596226

[21] Omar IA, Jayaraman R, Salah K, Yaqoob I, Ellahham S (2021) Applications of blockchain technology in clinical trials: review and open challenges. Arab J Sci Eng 46(4):3001–3015

[22] Katiyar D, Singhal S (2021) Blockchain technology in management of clinical trials: a review of its applications, regulatory concerns and challenges. Mater Today Proceed 47:198–206. 10.1016/j.matpr.2021.04.095

[23] Hang L, Chen C, Zhang L, Yang J (2022) Blockchain for applications of clinical trials: taxonomy, challenges, and future directions. IET Commun 16(20):2371–2393. 10.1049/cmu2.12488

[24] Zhang W (2022) Blockchain-based solutions for clinical trial data management: a systematic review. Metaverse Basic and Applied Research, 1, 17–17. 10.56294/mr202217

[25] Kumaravel P (2019) Clinical trials on blockchain. Achieving data integrity in clinical trials: utilizing blockchain technology. Symbiance Inc, PhUSE US Connect 2019, New Jersey, USA.

[26] Goldfarb NM (2018) Blockchain technology in clinical trials. Journal of Clinical Research Best Practices, 14(1).

[27] Bell L, Buchanan WJ, Cameron J, Lo O (2018) Applications of blockchain within healthcare. Blockchain in Healthcare Today. 10.30953/bhty.v1.8

[28] Sadiku MNO, Eze KG, Musa SM (2018) Block chain technology in healthcare. Int J Adv Sci Res Eng 4. 10.31695/IJASRE.2018.32723

[29] Nugent T, Upton D, Cimpoesu M (2016) Improving data transparency in clinical trials using blockchain smart contracts. F1000Research 5. 10.12688/f1000research.9756.1 10.12688/f1000research.9756.1 PMC5357027 28357041

[30] Dai H, Young HP, Durant TJS, Gong G, Kang M, Krumholz HM, Schulz WL, Jiang L (2018) Trialchain: a blockchain-based platform to validate data integrity in large, biomedical research studies. arXiv preprint arXiv:1807.03662. 10.48550/arXiv.1807.03662

[31] Kitchenham B, Hughes RT, Linkman SG (2001) Modeling software measurement data. IEEE Trans Software Eng 27(9):788–804. 10.1109/32.950316

[32] Cihan Ş, and Yılmaz N. Data extraction sheet, Google Drive, URL: https://tinyurl.com/2d4nltak

[33] Cihan Ş, and Yılmaz N. Matching articles with categories: Zenodo. https://zenodo.org/records/12624899

[34] Tang A, Han J, Chen P (2004) A comparative analysis of architecture frameworks. In 11th Asia-Pacific software engineering conference (pp. 640–647). IEEE. 10.1109/APSEC.2004.2

[35] IEEE Recommended Practice for architectural description for software-intensive systems, in IEEE Std 1471–2000 , vol., no., pp.1–30, 9 Oct, (2000). 10.1109/IEEESTD.2000.91944.

[36] Tovino SA (2020) Privacy and security issues with mobile health research applications. The Journal of Law, Medicine & Ethics, 48(1_suppl), 154–158. 10.1177/1073110520917041 10.1177/1073110520917041 32342741

[37] Tucker K, Branson J, Dilleen M, Hollis S, Loughlin P, Nixon MJ, Williams Z (2016) Protecting patient privacy when sharing patient-level data from clinical trials. BMC Med Res Methodol 16(1):5–14. 10.1186/s12874-016-0169-4 27410040 10.1186/s12874-016-0169-4 PMC4943495

[38] Yaqoob I, Salah K, Jayaraman R, Al-Hammadi Y (2022) Blockchain for healthcare data management: opportunities, challenges, and future recommendations. Neural Comput Appl 1–16:11475–11490. 10.1007/s00521-020-05519-w

[39] Wilkinson MD, Dumontier M, Aalbersberg IJ, Appleton G, Axton M, Baak A, Mons B (2016) The FAIR guiding principles for scientific data management and stewardship. Scientific data 3(1):1–9. 10.1038/sdata.2016.18 10.1038/sdata.2016.18 PMC4792175 26978244

[40] Sinaci AA, Núñez-Benjumea FJ, Gencturk M, Jauer ML, Deserno T, Chronaki C, Parra-Calderón CL (2020) From raw data to FAIR data: the FAIRification workflow for health research. Methods Inform Med 59(S 01):e21-e32. 10.1055/s-0040-1713684 10.1055/s-0040-1713684 32620019

[41] Zhang P, Walker MA, White J, Schmidt DC, and Lenz G (2017) Metrics for assessing blockchain-based healthcare decentralized apps. In 2017 IEEE 19th international conference on e-health networking, applications and services (Healthcom) (pp. 1–4). IEEE. 10.1109/HealthCom.2017.8210842

[42] Tatineni S (2022) Integrating AI, Blockchain and cloud technologies for data management in healthcare. J Computer Eng Technol (Jcet), 5(01).

[43] Singh LK, Khanna M (2023) Introduction to artificial intelligence and current trends. Innovations in Artificial Intelligence and Human-Computer Interaction in the Digital Era. Academic Press, 31–66. 10.1016/b978-0-323-99891-8.00001-2

[44] Singh LK, Khanna M, Garg H, Singh R (2024) Efficient feature selection based novel clinical decision support system for glaucoma prediction from retinal fundus images. Med Eng Phys 123:104077. 10.1016/j.medengphy.2023.104077 38365344 10.1016/j.medengphy.2023.104077

[45] Panarello A, Tapas N, Merlino G, Longo F, Puliafito A (2018) Blockchain and iot integration: a systematic survey. Sensors 18(8):2575. 10.3390/s18082575 30082633 10.3390/s18082575 PMC6111515

[46] Wu D, Wang Y (2024) Revolutionizing healthcare information systems with blockchain. Front Digit Health 5:1329196. 10.3389/fdgth.2023.1329196 38274085 10.3389/fdgth.2023.1329196 PMC10808696

[47] Shalaby S, Abdellatif AA, Al-Ali A, Mohamed A, Erbad A, and Guizani M (2020) Performance evaluation of hyperledger fabric. In 2020 IEEE International Conference on Informatics, IoT, and Enabling Technologies (ICIoT) (pp. 608–613). IEEE. 10.1109/ICIoT48696.2020.9089614

[48] Uddin M, Memon MS, Memon I, Ali I, Memon J, Abdelhaq M, Alsaqour R (2021) Hyperledger fabric blockchain: secure and efficient solution for electronic health records. Comput Mater Contin 68(2):2377–2397. 10.32604/cmc.2021.015354

[49] Ettaloui N, Arezki S, and Gadi T (2023) An overview of blockchain-based electronic health records and compliance with GDPR and HIPAA. Data and Metadata, 2, 166–166. 10.56294/dm2023166

[50] Canham S, Ohmann C (2016) A metadata schema for data objects in clinical research. Trials 17(1):1–11. 10.1186/s13063-016-1686-5 27881150 10.1186/s13063-016-1686-5 PMC5122021

[51] Ammann N, Nielsen LH, Peters CS, and de Smaele TM (2019) Datacite metadata schema for the publication and citation of research data. 10.5438/001

[52] Da Silva AR (2015) Model-driven engineering: a survey supported by the unified conceptual model. Comput Lang Syst Struct 43:139–155. 10.1016/j.cl.2015.06.001

[53] Beydoun G, Low G, Henderson-Sellers B, Mouratidis H, Gomez-Sanz JJ, Pavon J, Gonzalez Perez C (2009) FAML: a generic metamodel for MAS development. IEEE Trans Software Eng 35(6):841–863. 10.1109/TSE.2009.34

[54] Othman SH, Beydoun G, Sugumaran V (2014) Development and validation of a disaster management metamodel (DMM). Inf Process Manage 50(2):235–271. 10.1016/j.ipm.2013.11.001

[55] Yılmaz N, Tarhan AK (2023) Matching terms of quality models and meta-models: toward a unified meta-model of OSS quality. Software Qual J 31(3):721–773. 10.1007/s11219-022-09603-3

[56] Yılmaz N and Kolukısa A (2024) Quality evaluation meta-model for open-source software: multi-method validation study. Software Quality Journal. 10.1007/s11219-023-09658-w

[57] Cihan Ş, Yılmaz N (2024) Expert features consulted on the conceptual model. Zenodo. https://zenodo.org/records/13932035

[58] Cihan Ş, Yılmaz N (2024) A list of questions for obtaining feedback from experts. Zenodo. https://zenodo.org/records/12624582

[59] Yin RK (2018) The case study research and applications. Sage

